# Correction: The force at the implant cannot be assessed by the mallet force–Unless supported by a model

**DOI:** 10.1371/journal.pone.0332707

**Published:** 2025-09-17

**Authors:** Peter J. Schlieker, Michael M. Morlock, Gerd Huber

In Table 2, the damping parameter for the impactor tip has incorrect unit. The correct unit should be 0.24 Ns/mm. Please see the correct Table 2 here.

**Table 2:** Parameters of the numerical model including the results of the parameter estimation.

Object	Mass [kg]	Stiffness [N/mm]	Damping [Ns/mm]	Miscellaneous
Impactor	0.508	85 x 10^3^		
Load cells	0.137	2.3 x 10^6^		
Impactor tip	0.084	13 x 10^3^	0.24	
Transition between impactor tip and head				50 x 10^-6^ m
Femoral head	0.062			
Friction inside head taper junction				µ_kinetic_ = 0.49 µ_static_ = 0.79
Normal force of head taper junction				5.0 N 140 x 10^9^ N/m^2^
Stem taper replica	0.043	130 x 10^3^		
Analogous model of the responding tissue	0.52* and 0.89*	0.6 to 5.0	10% of stiffness	

* including load cell.
